# Regional ^18^F-fluoromisonidazole PET images generated from multiple advanced MR images using neural networks in glioblastoma

**DOI:** 10.1097/MD.0000000000029572

**Published:** 2022-07-29

**Authors:** Jianhua Qin, Yu Tang, Bao Wang

**Affiliations:** a School of Medicine, Qingdao University, Qingdao, P. R. China; b Department of Radiology, Rizhao Central Hospital, Rizhao, P. R. China; c Department of Radiology, Qilu Hospital of Shandong University, Jinan, P. R. China.

**Keywords:** ^18^F-FMISO, glioblastoma, hypoxia, MRI, neural network

## Abstract

Generated ^18^F-fluoromisonidazole (^18^F-FMISO) positron emission tomography (PET) images for glioblastoma are highly sought after because ^18^F-FMISO can be radioactive, and the imaging procedure is not easy. This study aimed to explore the feasibility of using advanced magnetic resonance (MR) images to generate regional ^18^F-FMISO PET images and its predictive value for survival.

Twelve kinds of advanced MR images of 28 patients from The Cancer Imaging Archive were processed. Voxel-by-voxel correlation analysis between ^18^F-FMISO images and advanced MR images was performed to select the MR images for generating regional ^18^F-FMISO images. Neural network algorithms provided by the MATLAB toolbox were used to generate regional ^18^F-FMISO images. The mean square error (MSE) was used to evaluate the regression effect. The prognostic value of generated ^18^F-FMISO images was evaluated by the Mantel-Cox test.

A total of 299 831 voxels were extracted from the segmented regions of all patients. Eleven kinds of advanced MR images were selected to generate ^18^F-FMISO images. The best neural network algorithm was Bayesian regularization. The MSEs of the training, validation, and testing groups were 2.92E-2, 2.9E-2, and 2.92E-2, respectively. Both the maximum Tissue/Blood ratio (*P* = .017) and hypoxic volume (*P* = .023) of the generated images were predictive factors of overall survival, but only hypoxic volume (*P* = .029) was a predictive factor of progression-free survival.

Multiple advanced MR images are feasible to generate qualified regional ^18^F-FMISO PET images using neural networks. The generated images also have predictive value in the prognostic evaluation of glioblastoma.

## 1. Introduction

Glioblastoma multiforme (GBM) is the most malignant primary tumor of the brain, with a high mortality and poor prognosis.^[1]^ Radiation treatment is a common therapy for GBM patients after surgical resection. However, many patients did not benefit from the treatment because of radiation resistance of GBM. Hypoxia plays a vital role in radiation resistance.^[2]^

The strategy of radiation therapy could be improved by the identification of hypoxic subregions of GBM because a higher radiation dose could be delivered to the hypoxic subregions to overcome radiation resistance.^[3]^ Noninvasive and reliable hypoxic imaging has always been treated as an appropriate approach to evaluate the hypoxic conditions of tumors.^[4]^ ^18^F-Fluoromisonidazole (^18^F-FMISO) is a positron emission tomography (PET) imaging agent that selectively binds to hypoxic tissues. ^18^F-FMISO uptake has been investigated because of correlation with polarographic oxygen electrodes, which is the gold standard in evaluating hypoxia.^[5]^ Previous studies have confirmed the value of ^18^F-FMISO PET in evaluating hypoxic conditions and prognosis in GBM.^[4,6]^

Although ^18^F-FMISO PET is quite helpful in identifying the hypoxic region, the radioactivity of PET examination cannot be avoided.^[4]^ In addition, preparation of ^18^F-FMISO was not easy, and patient blood should be collected to investigate the average blood activity to produce a pixel-level tissue-to-blood ratio.^[7]^ These disadvantages may restrict the widespread use of ^18^F-FMISO PET in clinical practice. Generating images giving the same information as ^18^F-FMISO PET images with other nonradioactive imaging methods with easy accessibility is always desired and meaningful. Dynamic contrast-enhanced perfusion-weighted imaging (DCE-PWI) and dynamic susceptibility contrast perfusion-weighted imaging (DSC-PWI) could reflect the permeability and hemodynamic characteristics of abnormal vasculature in GBM.^[8,9]^ An apparent diffusion coefficient (ADC) map has been validated to be correlated with tumor cell density, and a fractional anisotropy map can evaluate the conditions of white matter damage.^[10]^ These pathophysiological processes reflected by the above advanced MR images are associated with hypoxia in GBM.^[11–13]^ Generating images giving the same information as ^18^F-FMISO PET images from these advanced MR images seemed feasible.

Neural networks are a subset of machine learning and are at the heart of deep learning algorithms. Their name and structure are inspired by the human brain, mimicking the way that biological neurons signal to one another. Neural networks are comprised of node layers, containing an input layer, one or more hidden layers, and an output layer. Each node, or artificial neuron, connects to another and has an associated weight and threshold. They have become popular and powerful tools in image translation.^[14,15]^ This technique may be an appropriate tool for generating images that provide the same information as ^18^F-FMISO PET images. Therefore, this study aimed to explore the feasibility of generating regional ^18^F-FMISO PET images with multiple advanced MR images via neural network methods and their predictive value for survival.

## 2. Materials and Methods

### 2.1. Study population

**Figure 1.**
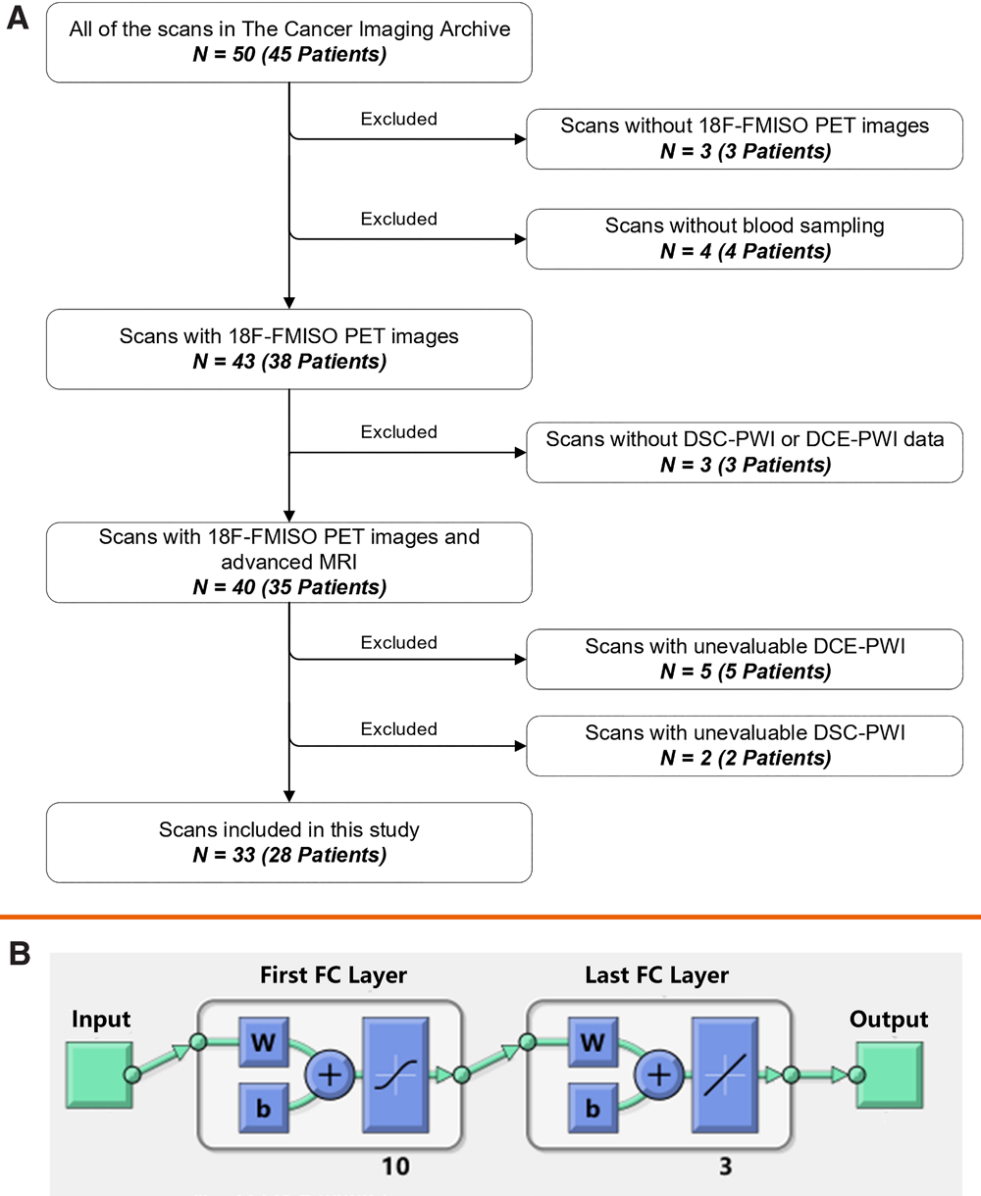
Flowchart of patient inclusion and diagram of neural network. (A) Patients inclusion and exclusion. (B) Input: this layer corresponds to the predictor data. First FC layer: this layer has 10 outputs by default; ReLU activation function is applied to the first fully connected layer. Final FC layer, this layer has one output. Output, this layer corresponds to the predicted response values. N in part A represents the number of scans. DCE-PWI = dynamic contrast-enhanced perfusion-weighted imaging, DSC-PWI = dynamic susceptibility contrast perfusion-weighted imaging, FC = fully connected, FMISO = fluoromisonidazole, PET = positron emission tomography, ReLU = rectified linear unit.

All imaging and clinical data were collected from the ACRIN-FMISO-Brain dataset in The Cancer Imaging Archive. According to the description of the dataset, the patients in this dataset had some residual tumor (as determined by the treating physician) after surgery but before radiotherapy and chemotherapy, based on postcontrast T1WI or T2 fluid-attenuated inversion recovery (T2-FLAIR) imaging, although the minimum amount was not specified. Of the 45 patients in the ACRIN-FMISO-Brain dataset, 17 patients were excluded because of no evaluable ^18^F-FMISO PET scans or advanced MR images. Finally, 28 patients with 33 scans were included in this study. Figure 1 demonstrates how sample size was derived. The summarized clinical characteristics of the study population are provided in Table 1. The workflow of this study is provided in the Supplementary Files, http://links.lww.com/MD/G930.

**Table 1:** Participant characteristics.

Characteristics	Descriptive information
Total number	28
Age, y, mean ± SD	56.3 ± 8.8
Gender, n, male, (%)	17 (60.7%)
Time from surgery to FMISO PET, d, median [IQR]*	1 [0–4]
Time from surgery to MRI, d, median [IQR]†	1.5 [0–6]
Residual tumor size, cc, [IQR]	4.6 [1.3–11.8]
Survival	
OS, months, median, [IQR]	15 [9.8–21]
Alive over 1 year, n (%)	15 (53.6%)
PFS, months, median, [IQR]	9 [6–12]
Progression-free over 9 months, n, (%)	11 (39.3%)

This retrospective study received approval from the hospital’s ethical committee. Written informed consent was not required for this study because the data were acquired from The Cancer Imaging Archive. All experiments were performed in compliance with the Declaration of Helsinki.

### 2.2. MR examination and image acquisition

MR imaging (MRI) and ^18^F-FMISO PET were performed separately during a small interval. All sites followed a standardized acquisition protocol for these scans. (e.g., see http://www.acrin.org/Portals/0/Protocols/6684/ACRIN6684_Amend7_012412_master_ ForOnline.pdf for full details). Only MRI data consisting of precontrast T1WI, T2-FLAIR images, T1 mapping, DSC-PWI, DCE-PWI, DTI, and postcontrast T1WI were used in this study.

### 2.3. Generation of Quantitative MR images

First, T1 mapping, DSC-PWI, DCE-PWI, and DTI were used to generate advanced MR images. All the raw data were transferred to dedicated workstations, and postprocessing was performed with commercial software (NordicICE, version 4.0.6; NordicNeuroLab, Bergen, Norway) by an experienced neuroradiologist (over 15 years). Detailed processing information is provided in the Supplementary Files, http://links.lww.com/MD/G930.

Second, each of the generated maps was normalized by dividing the mean value of the pons. This is necessary for advanced MR images and can be considered as the normalization step in individual level. Finally, 12 kinds of normalized advanced MR images, including T1 mapping, cerebral blood flow (CBF) map, cerebral blood volume (CBV) map, contrast agent transfer constant *K^trans^* and *K^ep^*, vascular fraction (*V_p_*), extravascular fraction (*V*_*e*_), time to peak map, peak map, area under the curve map, ADC, and fractional anisotropy maps, were used to generate ^18^F-FMISO PET images.

### 2.4. Processing of Quantitative MR images and FMISO image

The FMISO image data should be normalized by the average blood activity to produce pixel-level tissue-to-blood ratio (T/B) values for all image slices. In addition, the twelve kinds of advanced MR images and FMISO images have a similar scale.

First, they were registered to postcontrast T1WI images in noncommercial software (ITK-SNAP, Version 3.6.0; www.itksnap.org). Then, the moving images (advanced MR maps and ^18^F-FMISO PET images) were resliced into the space of the postcontrast T1WI images with linear interpolation. At last, smoothing was performed on all the resliced images with a full width at half maximum of [8,8,8]. In addition, T2-FLAIR images were also processed as above steps.

### 2.5. Segmentation for further analysis

Segmentations were manually performed on ITK-SNAP by an experienced radiologist (over 10 years). The segmentations should cover the enhancing lesions in postcontrast T1WI (after normalization and smoothing) and high-intensity peritumoral region areas in the processed T2-FLAIR images (after normalization and smoothing). Hypoxia may be in peritumoral regions.^[16]^ In addition, another neuroradiologist (with >15 years of experience) confirmed the segmentations.

### 2.6. Training the fitting model by neural networks

Only the voxels within the segmentation were used for further analysis. The values of voxels were extracted in MATLAB (version 2020a, Mathwork, Inc., Natick, MA, USA). First, the matrix data form within the segmentation of each scan was transformed to the vector data form within a fixed arrangement principle provided by MATLAB. Second, the values of all scans were combined into a matrix dataset (the column represents the variables, and the rows represent the scans). As there are 12 kinds of advanced MR images and 1 FMISO PET image, therefore, there are 13 columns in total. Advanced MR values were treated as numeric predictors and FMISO PET value was treated as response variable. The data of each column (voxels values from the same advanced MR images) would be rescaled into 0 to 1, and it is considered as the normalization step in pixels level. Normalization at individual level and pixel level would help us improve the performance of training models. Voxel-by-voxel correlation between FMISO images and 12 advanced MR images was evaluated to explore the relationships between FMISO images and advanced MR images.

Two-layer feed-forward networks were used to train the fitting model in this study. The voxel values of 12 advanced MR images were treated as predictor data and those of FMISO images were treated as response values. The first fully connected layer of the neural network has a connection from the network input (predictor data), and each subsequent layer has a connection from the previous layer. Each fully connected layer multiplies the input by a weight matrix and then adds a bias vector. An activation function follows each fully connected layer, excluding the last. The final fully connected layer produces the network’s output, namely predicted response values.

The dataset was transported to the *Neural Net Fitting App* in MATLAB, among which 70 percent, 15 percent, and 15 percent of the dataset were used for training, validation, and testing, respectively. The number of hidden neurons was 10. Three training algorithms, Levenberg-Marquardt, Bayesian regularization, and scaled conjugate gradient, were selected separately to train the data. Evaluate the model at each iteration by using the validation set. By default, the training process ends early if the validation loss is greater than or equal to the minimum validation loss computed so far, 6 times in a row. The mean square error (MSE) was used to select the best fitting algorithm. All the network computations were performed on a GPU workstation (Nvidia Tesla P4 GPU with Intel Xeon[R] central processing unit [CPU] E5-2667 v4 at 3.20). The neural network diagram is shown in Figure 1.

### 2.7. Analysis of generated regional FMISO image

The predicted FMISO values of each patient were in the vector data form. Therefore, to generate regional FMISO images, the vector data form was reorganized to the matrix data form within the same arrangement principle provided by MATLAB. To quantitate hypoxia in each tumor region on the generated regional FMISO image, the pixel with the maximum T/B value (TB_max_) and the hypoxic volume (HV) were determined. The HV was determined as the volume of pixels in the tumor ROI with a T/B ratio > 1.2. This cutoff value was previously shown to indicate significant hypoxia.^[17]^ HV determines the spatial extent of hypoxia in a tumor, whereas TB_max_ reports the severity of hypoxia. Both HV and TB_max_ have been shown to be independent predictors of outcome in brain cancer.^[16]^ The root mean squared error was used to evaluate the accuracy of the predicted TB_max_ and HV.

### 2.8. Statistical analysis

Pearson correlation analysis was performed to investigate the relationship between FMISO images and 12 kinds of advanced MR images. Survival events were defined as death from any cause for overall survival (OS) and as disease progression for progression-free survival (PFS). OS was calculated from the time of histologic diagnosis of the tumor, and PFS was calculated from the time of resection to tumor progression using the Kaplan-Meier method. The prognostic values of HV and TB_max_ were evaluated by the log-rank (Mantel-Cox) test. A *P* value of < .05 was considered statistically significant. For multiple tests, Bonferroni correction was used. All tests were performed using MATLAB.

## 3. Results

### 3.1. Correlations between advanced MR images and FMISO image

In total, 299 831 voxels were extracted from all the patients, and the values of all modalities were recorded. All advanced MR images excluding *K*_*ep*_ were significantly correlated with FMISO images (all *P* < .001), among which normalized CBV had the highest correlation efficiency. Therefore, *K*_*ep*_ was not used for further image generation. The results of the correlation analysis between advanced MR images and FMISO images are shown in Table 2.

**Table 2:** Correlations between advanced MR images and FMISO image.

	r	95% CI	R squared	*P* value
ADC	−0.252	[−0.256 to −0.249]	0.064	<.001
AUC	0.183	[0.179 to 0.186]	0.033	<.001
nCBF	0.35	[0.347 to 0.353]	0.123	<.001
nCBV	0.418	[0.415 to 0.421]	0.174	<.001
FA	0.082	[0.078 to 0.085]	0.007	<.001
*K^trans^*	0.152	[0.149 to 0.156]	0.023	<.001
*K*_*ep*_	−0.002	[−0.005 to 0.002]	2.87E-6	.35
Peakmap	0.175	[0.171 to 0.178]	0.03	<.001
T1	0.178	[0.174 to 0.181]	0.032	<.001
TTP	0.068	[0.064 to 0.071]	0.005	<.001
*V*_*e*_	0.134	[0.13 to 0.137]	0.018	<.001
*V*_*p*_	0.268	[0.264 to 0.271]	0.072	<.001

### 3.2. Fitting performance of neural networks

**Figure 2.**
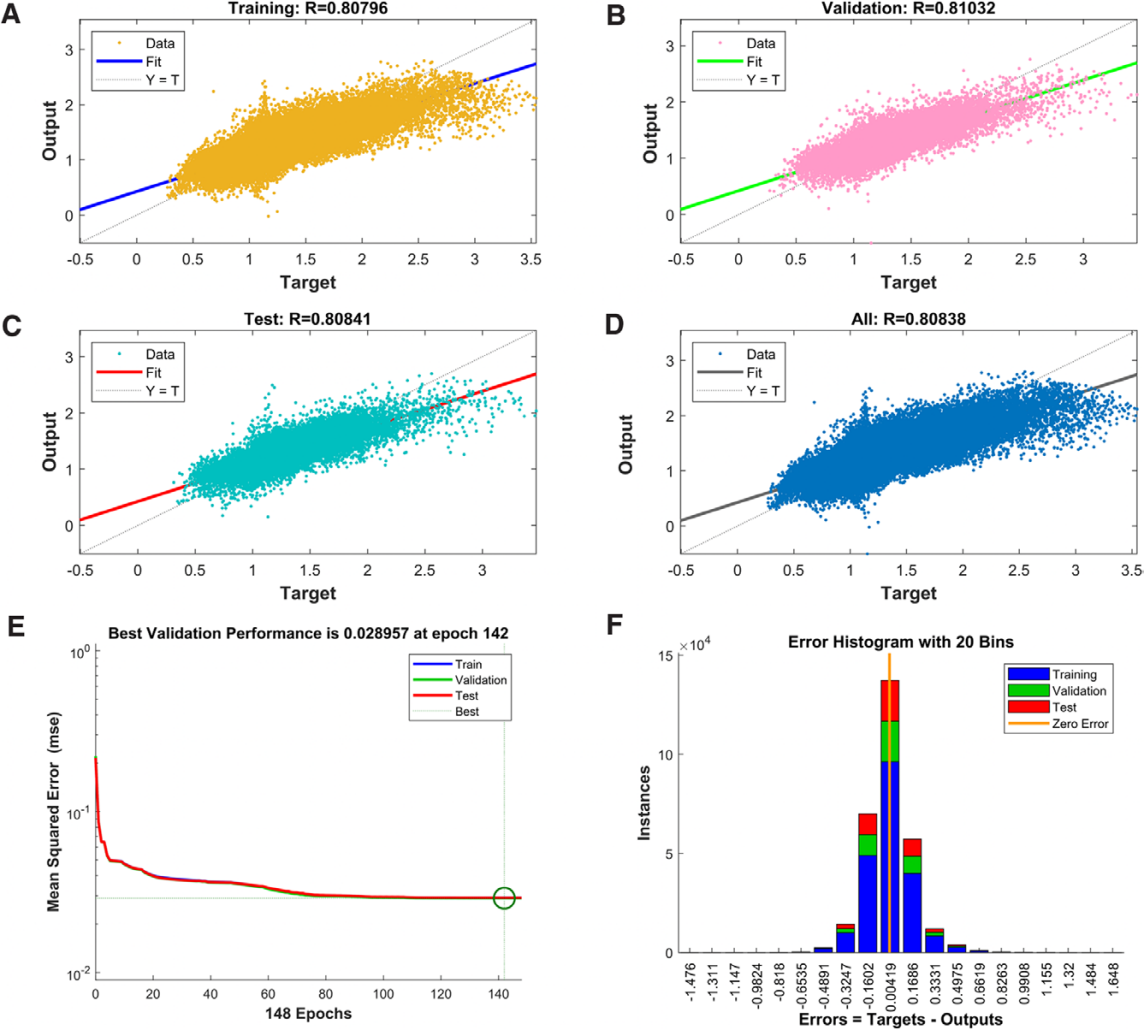
Detailed results of Bayesian regularization algorithms. (A) Regression performance of training data; (B) regression performance of validation data; (C) regression performance of test data; (D) regression performance of all datasets; (E) best validation performance; (F) error histogram with 20 bins (errors = ground truths - predictive values).

**Figure 3.**
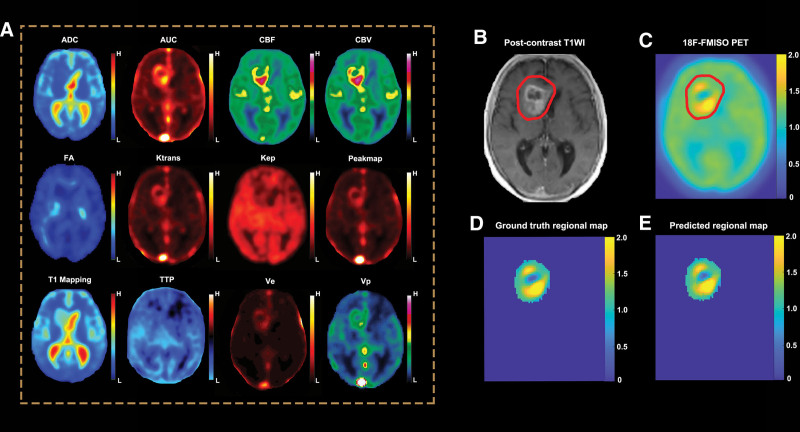
Demonstration of one case with obvious hypoxic conditions. An old female patient with newly diagnosed GBM. (A) Twelve kinds of advanced MR images of these patients; (B) postcontrast T1WI and region of interest (with the red line); (C) whole ^18^F-FMISO PET image and the same region of interest; (D) ground-truth regional ^18^F-FMISO PET; (E) generated regional ^18^F-FMISO PET, which has a high concordance with ground truth. ADC = apparent diffusion coefficient, AUC = area under the curve, FA = fractional anisotropy, FMISO = fluoromisonidazole, GBM = glioblastoma multiforme, MR = magnetic resonance, nCBF = normalized cerebral blood flow, nCBV = normalized cerebral blood volume, PET = positron emission tomography, *V*_*e*_ = extravascular fraction, *V*_*p*_ = vascular fraction.

**Figure 4.**
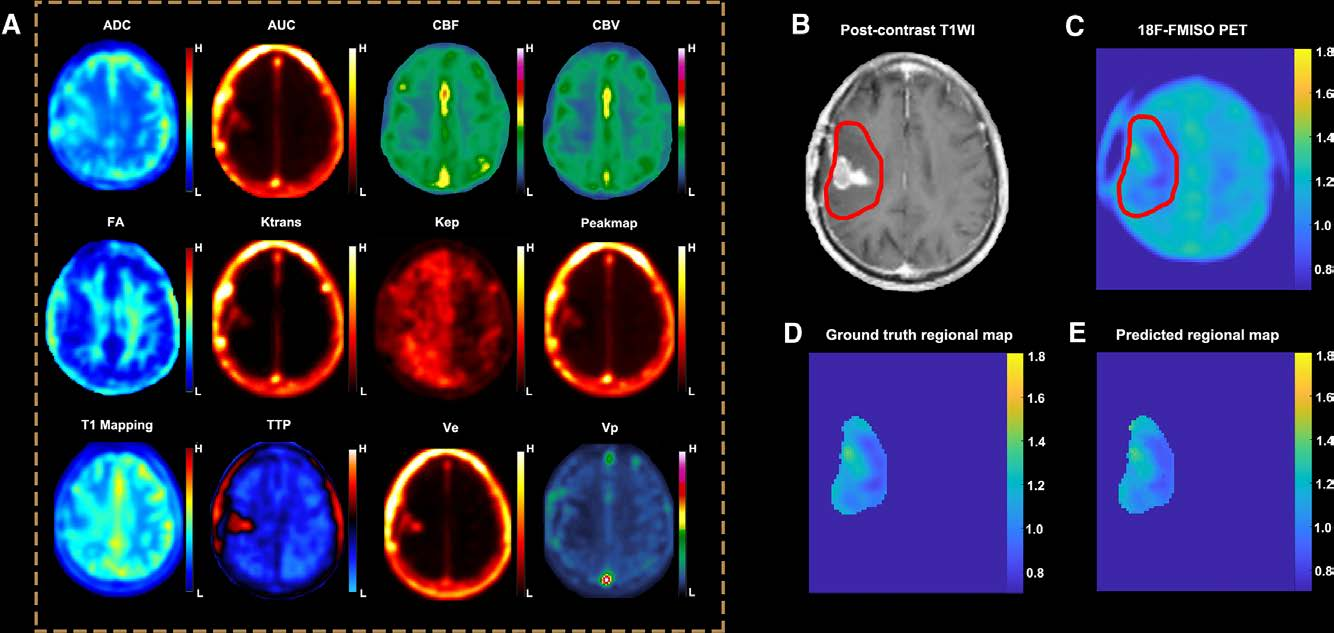
Demonstration of one case with limited hypoxic conditions. An old male patient with residual GBM after surgical resection. (A) Twelve kinds of advanced MR images of these patients; (B) Postcontrast T1WI and region of interest (with the red line); (C) Whole ^18^F-FMISO PET image and the same region of interest; (D) ground-truth regional ^18^F-FMISO PET; (E) Generated regional ^18^F-FMISO PET, which has a high concordance with ground truth. ADC = apparent diffusion coefficient, AUC = area under the curve, FA = fractional anisotropy, FMISO = fluoromisonidazole, GBM = glioblastoma multiforme, MR = magnetic resonance, nCBF = normalized cerebral blood flow, nCBV = normalized cerebral blood volume, PET = positron emission tomography, *V*_*e*_ = extravascular fraction, *V*_*p*_ = vascular fraction.

The training algorithm of Bayesian regularization^[18]^ achieved the highest fitting performance, and the MSEs of the training, validation, and testing groups of Bayesian regularization were 2.92 × 10E-2, 2.9E-2, and 2.92E-2, respectively. The results of 3 training algorithms are summarized in Table 3. The detailed results of the Bayesian regularization algorithms are displayed in Figure 2. One case with obvious hypoxic conditions is shown in Figure 3, and another case with limited hypoxic conditions is shown in Figure 4.

**Table 3:** Mean squared error of 3 neural network algorithms.

	Training	Validation	Test
Bayesian regularization	2.92E-2	2.90E-2	2.92E-2
Levenberg-Marquardt	3.01E-2	3.0E-2	3.02 E-2
Scaled conjugate gradient	5.15E-2	5.06E-2	5.25E-2

### 3.3. Accuracy of predicted TB_max_ and HV and their prognostic value

The ground-truth value of TB_max_ was 2.11 ± 0.79, and the predicted value of TB_max_ was 2.09 ± 0.73. The RMSE between them was 0.077. The ground-truth value of HV was 13.18 ± 11.52, and the predicted value of HV was 13.21 ± 11.57. The root mean squared error between them was 0.054.

**Figure 5.**
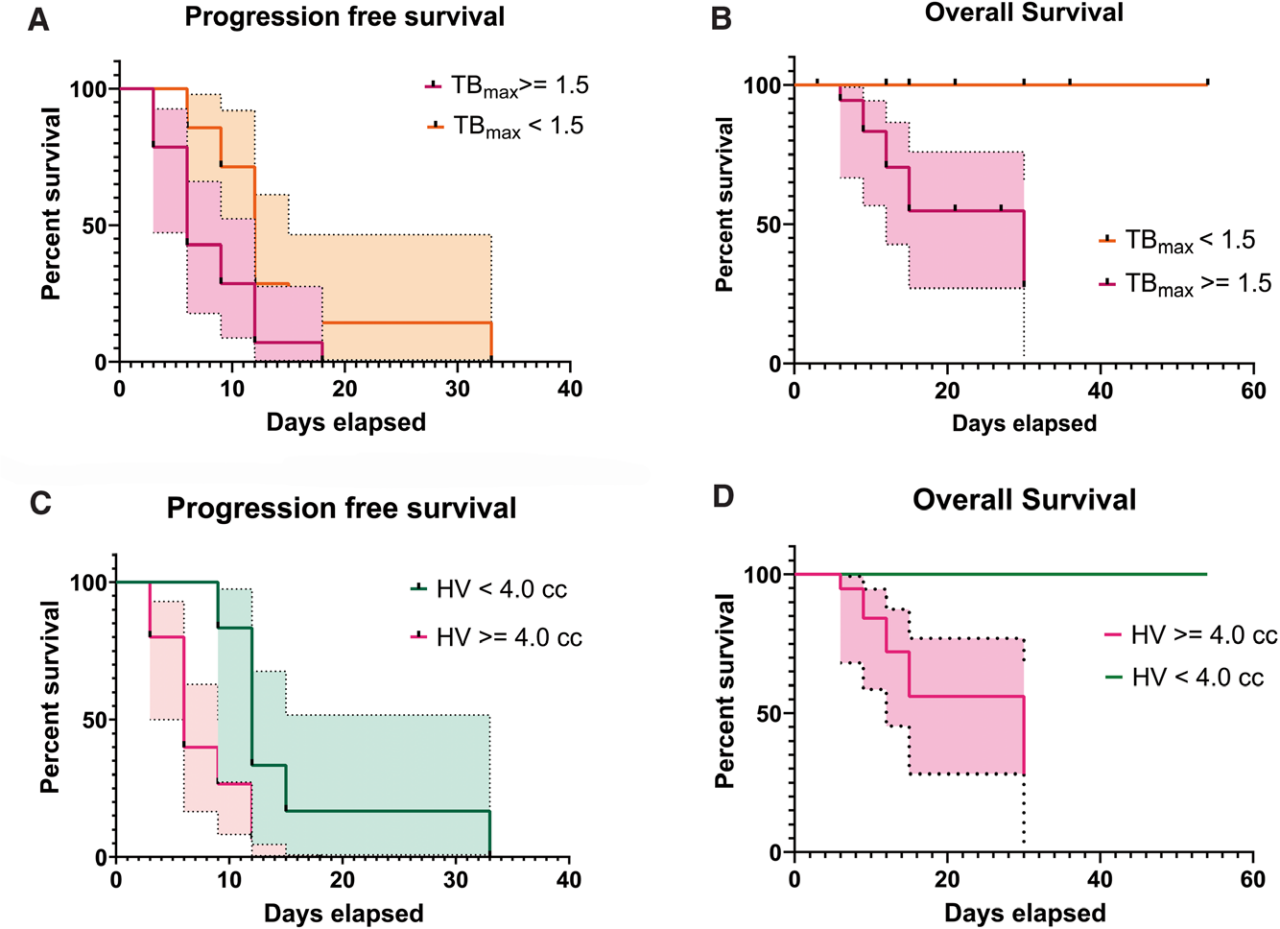
Log-rank (Mantel-Cox) test results of TB_max_ and HV. (A) PFS in 2 groups with different TB_max_ was not significantly different (*P* = .064); (B) OS in 2 groups with different TB_max_ was significantly different (*P* = .017); (C) PFS in 2 groups with different HV was significantly different (*P* = .029); (D) OS in 2 groups with different HV was significantly different (*P* = .023). HV, hypoxic volume, OS = overall survival, PFS = progression-free survival, TB_max_, the maximum tissue-to-blood ratio.

Previous study has found that an HV value of 4.0 cc and TB_max_ of 1.5 could be selected as the cutoff values to separate the patients into good and poor prognostic groups.^[19]^ The 2 groups separated by ground-truth TB_max_ or HV were the same as those separated by predicted TB_max_ or HV. The log-rank (Mantel-Cox) test showed that both TB_max_ (*P* = .017) and HV (*P* = .023) were predictive factors of OS. HV (*P* = .029) was a predictive factor of PFS, but TB_max_ was not (*P* = .064). The Log-rank (Mantel-Cox) is shown in Figure 5.

## 4. Discussion

^18^F-FMISO PET is a useful but radiotoxic and inconvenient method to evaluate the hypoxic condition and prognosis of GBM. This article explored the feasibility of generating regional ^18^F-FMISO PET images from multiple advanced MR images via neural network fitting methods. The results showed that this noninvasive method could provide appropriate regional ^18^F-FMISO PET images for clinical hypoxic and prognostic evaluation.

### 4.1. Association between MRI parameters and the degree of hypoxia

Gerstner et al^[20]^ reported that there was a moderate positive correlation between normalized CBF (nCBF) and HV. In addition, Bekaert et al^[21]^ reported a significant correlation between rCBV and the degree of hypoxia (HV and SUV_max_). Recently, Keven et al^[22]^ found a tight association between hypoxia and angiogenesis in GBM. These findings suggested that advanced MR parameters, especially perfusion parameters, could reflect the degree of hypoxia. Our results showed that all advanced MR images excluding *K*_*ep*_ were significantly correlated with the uptake of ^18^F-FMISO. The results indicate that changes in the permeability and hemodynamic condition of tumor vessels may take part in the process of hypoxia in GBM, as well as the cellular density and damage to white matter.^[4]^

However, the abnormal vasculature in GBM is immature and inefficient to deliver enough oxygen and nutrients to tumor cells, and hypoxic conditions upregulate the expression of vascular endothelial growth factors, which further promotes aggressive and immature angiogenesis and leads to worse hypoxia.^[23,24]^ A recent animal study also confirmed the correlation between ^18^F-FMISO uptake and blood flow, blood volume, and the metabolic rate of oxygen,^[25]^ which could explain why nCBV, nCBF, and *V*_*p*_ had a higher correlation with the uptake of ^18^F-FMISO than other parameters. However, a higher cellular density means more oxygen occupation than under normal conditions,^[10]^ which may explain why ADC values also had a higher correlation. Other quantitative parameters with an absolute *R* value < 0.2 may indicate that the physiological changes associated with them play less important roles in the development of hypoxic environments.

### 4.2. Potential influence on treatment strategy

PET could provide functional information by visualizing the metabolism of the cell. In radiation therapy for cancer, the tumor oxygen concentration is an important factor that greatly affects the therapeutic effects.^[3,7]^ The radiation sensitivity of cells is thus reduced under hypoxic conditions below a certain oxygen concentration threshold.^[3,21]^ In addition to causing radioresistance, tumor hypoxia also interferes with the cytotoxic activities of many types of chemotherapy, a phenomenon known as chemoresistance.^[26]^ Evaluation of the hypoxic subregion of tumors would help adjust the treatment strategy. For example, to achieve better disease control, a higher dose of radiation would be delivered to the tumor if it had a severely hypoxic environment.

### 4.3. Potential influence on predicting prognosis

Gerstner et al^[20]^ and Bekaert et al^[21]^ reported a different predictive value of ^18^F-FMISO PET in PFS and OS.^[20,21]^ This difference may result from the various criteria for separating the 2 groups with different prognoses. Gerstner et al^[20]^ found that only SUV_max_ could predict the one-year OS of patients with GBM. Bekaert et al^[21]^ found that patients with no uptake of ^18^F-FMISO had a longer PFS and OS than those without ^18^F-FMISO. In this study, using the same follow-up results but a different cutoff value of TB_max_ and HV from Gerstner et al^[20]^, we found that both TB_max_ and HV could be predictors of patient prognosis. These findings may reveal the importance of the cutoff value in evaluating prognosis.

However, the ground-truth TB_max_ and HV had a small variance compared to the predicted values, and the 2 prognostic groups separated by ground-truth TB_max_ or HV were the same as those separated by predicted TB_max_ or HV. This result indicates the validity and feasibility of the predicted TB_max_ or HV. However, although controversy exists regarding the prognostic value, the predicted TB_max_ or HV could still provide some prognostic value to clinicians. However, whether the spatial extent of hypoxia (HV) plays a more vital role than the severity of hypoxia (TB_max_) when predicting prognosis still needs to be explored in a large study.

Some limitations should be addressed here. Only a limited number of patients were included in this study because we could only obtain these valuable data from the public dataset. To guarantee the training efficiency of input data under the condition of limited training data, on the one hand, instead of using image-to-image translation/generation, we used a voxel-to-voxel value regression method to achieve data augmentation; on the other hand, we made the regional part of brain images the input of machine learning to only generate regional ^18^F-FMISO images to improve the data quality.

In conclusion, we developed a feasible approach to generating regional ^18^F-FMISO images from multiple advanced MR images to evaluate the hypoxic condition and prognosis of GBM, making the process of assessing tumoral hypoxia nonradiotoxic and noninvasive, facilitating the adjustment of treatment strategy and prognosis prediction.

## Author contributions

Guarantor of integrity of the entire study: Bad Wang

Study concepts and design: Bao Wang & Jianhua Qin

Literature research: Yu Tang

Experimental studies/data analysis: Yu Tang & Jianhua Qin

Statistical analysis: Bao Wang

Manuscript preparation: Jianhua Qin

Manuscript editing: Bao Wang

## Supplementary Material



## Footnotes

* Multiple FMISO PET scans in one patient were all recorded.

† Multiple MRI scans in one patient were all recorded.
