# Supplementary material for: Regional 18F-fluoromisonidazole PET images generated from multiple advanced MR images using neural networks in glioblastoma
Source: Medicine (Baltimore). 2022 Jul 29;101(30):e29572. doi: 10.1097/MD.0000000000029572 (PMC9333488; doi:10.1097/MD.0000000000029572)
Supplement: Supplementary file 1 [file medi-101-e29572-s001.pdf]

Supplementary files

1. The workflow of this study

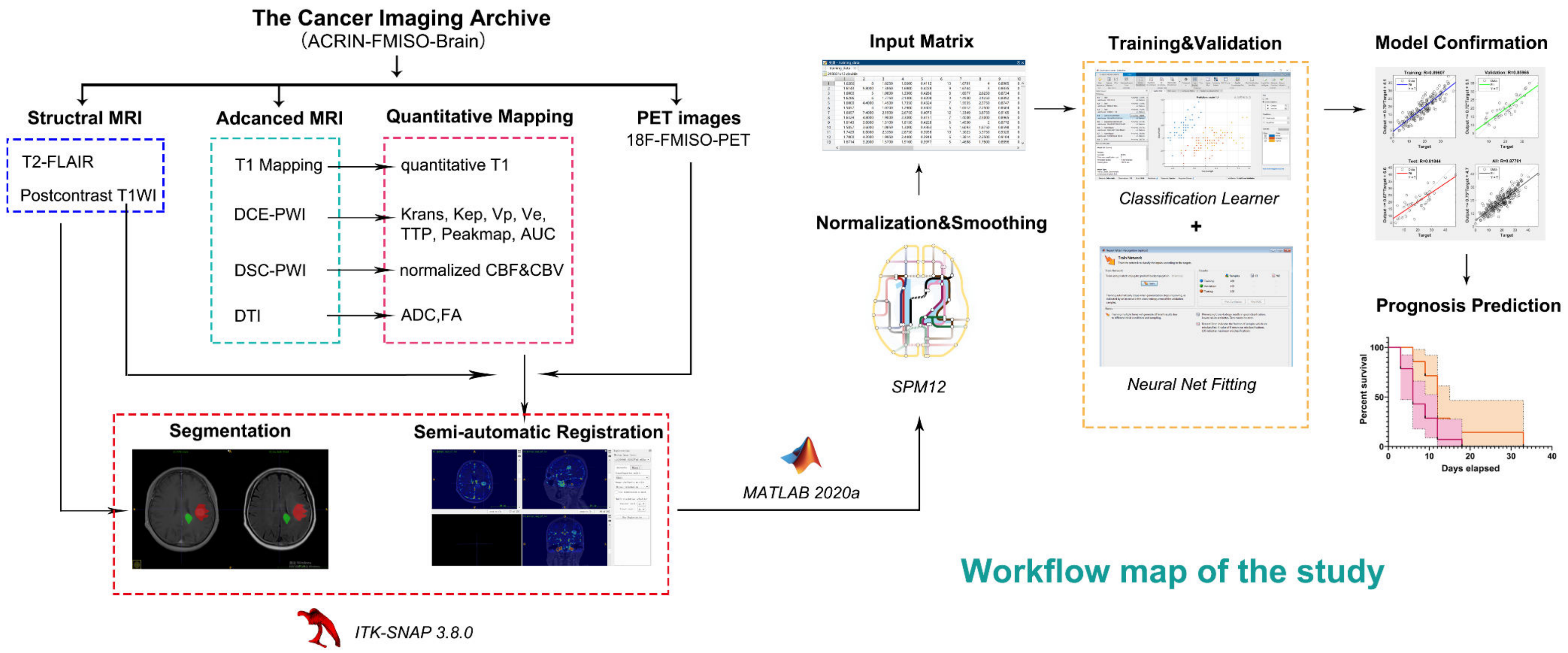

Workflow map of the study

2. The detailed processing information of quantitative MR images

T1 Mapping: five kinds of T1-weighted SPGR images with different flip angles (2, 10,15, 20, and 30 degrees, separately) were transferred to the T1 Mapping module. Noise threshold was automatically detected and motion correction was performed. Mono-exponential model was used for T1 Mapping curve fitting. Least-squares weighting was equal weighting for all datapoints.

DSC-PWI: the multiple phases of T2* imaging data were transferred to the Perfusion Module. Pre-bolus range and last images were manually selected. The signal conversion was signal intensity to delta R2. Noise threshold was automatically detected, and motion correction as well as temporal smoothing were performed during pre-process. Arterial input function (AIF) deconvolution analysis was with a type of standard singular value deconvolution, and based on relative blood volume from normalized area under the curve. Besides, contrast agent leakage correction was performed with Weisskoff method and positive T2 correction. Finally, normalized cerebral blood volume (CBV) and cerebral blood flow (CBF) maps were generated.

DCE-PWI: the multiple phases of T1-weighted SPGR images were transferred to the DCE Image Analysis Module. Pre-bolus range, last images and wash-in range were manually selected. The signal conversion was 1/T1 from SPGR. Noise threshold was automatically detected, and motion correction as well as temporal smoothing were performed during pre-process. The generated T1 Mapping was used for the baseline T1 calculation. For AIF determination, automatic detection with 5 pixels and 50 iterations was performed from the slice that cover the distribution of M1 part of the middle cerebral artery. If no appropriate AIF

was detected, the AIF would be manually selected by placing the region of interest (ROI) on the appropriate region. Extended Tofts model (3-parameter model) was used as kinetic model with AIF deconvolution. Hematocrit correction factor was 0.45. K^{trans}, K^{ep}, V_p, V_e, time to peak (TTP), Peakmap, area under the curve (AUC) map were generated.

DTI: the diffusion data with 32 gradient directions was transferred to the Diffusion Analysis Module. Noise threshold was automatically detected, and motion correction as well as spatial smoothing were performed during pre-process. Gradient directions matrix was manually selected according to the vendors. The FA and ADC maps were generated.
